# Early-Phase ^11^C-PiB PET in Amyloid Angiopathy-Related Symptomatic Cerebral Hemorrhage: Potential Diagnostic Value?

**DOI:** 10.1371/journal.pone.0139926

**Published:** 2015-10-06

**Authors:** Karim Farid, Young T. Hong, Franklin I. Aigbirhio, Tim D. Fryer, David K. Menon, Elizabeth A. Warburton, Jean-Claude Baron

**Affiliations:** 1 APHP, Hotel-Dieu Hospital, Department of Nuclear Medicine, Paris, France; 2 Dept of Nuclear Medicine, Martinique University Hospital, Fort-de-France, French West Indies; 3 Wolfson Brain Imaging Centre, Dept of Clinical Neurosciences, University of Cambridge, Cambridge, United Kingdom; 4 Division of Anesthesia, University of Cambridge, Cambridge, United Kingdom; 5 Stroke Research Group, Department of Clinical Neurosciences, University of Cambridge, Cambridge, United Kingdom; 6 Stroke Unit, Addenbrooke’s Hospital, Cambridge, United Kingdom; 7 INSERM U894, Centre Hospitalier Sainte Anne, Sorbonne Paris Cité, Paris, France; Nathan Kline Institute and New York University School of Medicine, UNITED STATES

## Abstract

Although late-phase (>35min post-administration) ^11^C-PiB-PET has good sensitivity in cerebral amyloid angiopathy (CAA), its specificity is poor due to frequently high uptake in healthy aged subjects. By detecting perfusion-like abnormalities, early-phase ^11^C-PiB-PET might add diagnostic value. Early-frame (1–6min) ^11^C-PiB-PET was obtained in 11 non-demented patients with probable CAA-related symptomatic lobar intracerebral haemorrhage (70±7yrs), 9 age-matched healthy controls (HCs) and 10 HCs <55yrs. There was a significant decrease in early-phase atrophy-corrected whole-cortex SUV relative to cerebellar vermis (SUVR) in the CAA vs age-matched HC group. None of the age-matched controls fell below the lower 95% confidence limit derived from the young HCs, while 6/11 CAA patients did (sensitivity = 55%, specificity = 100%). Combining both early- and late-phase ^11^C-PiB data did not change the sensitivity and specificity of late-phase PiB, but combined early- and late-phase positivity entails a very high suspicion of underlying Aβ-related clinical disorder, i.e., CAA or Alzheimer disease (AD). In order to clarify this ambiguity, we then show that the occipital/posterior cingulate ratio is markedly lower in CAA than in AD (N = 7). These pilot data suggest that early-phase ^11^C-PiB-PET may not only add to late-phase PiB-PET with respect to the unclear situation of late-phase positivity, but also help differentiate CAA from AD.

## Introduction

Cerebral Amyloid Angiopathy (CAA) is an increasingly frequent cause of intracerebral haemorrhage and the main cause of lobar intracerebral haemorrhage (LICH), and also contributes to cognitive and gait dysfunction in the elderly[1]. The hallmark of CAA is marked β-amyloid (Aβ) deposition within the wall of leptomeningeal and cortical arterioles, which is also a frequent *post mortem* finding in asymptomatic elderly subjects and in patients with Alzheimer’s disease (AD)[1]. Clinical and laboratory criteria have been proposed for the diagnosis of CAA. Although a definite diagnosis requires a full post-mortem examination, this is not useful in the clinical setting and accordingly less definite diagnostic degrees are widely employed in daily practice. Thus, the diagnosis of ‘probable CAA’ in symptomatic LICH (sLICH) as proposed in the validated Boston criteria[2] relies on a second LICH or the presence of strictly lobar microbleeds on T2* MRI, and no other cause identified.

^11^C-Pittsburgh compound B (PiB) has been developed as a PET ligand for imaging cerebral fibrillar β-amyloid (Aβ), using either kinetic modelling of the tissue pharmacokinetics over 60–90mins following tracer administration, or late-phase uptake images (typically >40min)[3,4]. Brain ^11^C-PiB-PET correlates with post-mortem fibrillar Aβ deposition extent in the brain associated with AD, but also with cerebrovascular Aβ deposits[3,5,6]. Amyloid PET might therefore directly detect vascular amyloid in living patients, which in turn could help towards diagnosing probable CAA in patients with sLICH not fulfilling the Boston criteria. Recently, we reported that in patients with sLICH a negative late-phase PiB scan ruled out CAA with 91% sensitivity, but had low specificity (55%) due to the frequent occurrence of high whole-cortex ^11^C-PiB binding in the cognitively healthy elderly[7], a well-established notion[8,9].

Improving the specificity of late-phase PiB-PET for the diagnosis of probable CAA by means of a second imaging variable would therefore be of clinical utility. One widely used imaging approach in neurodegenerative and age-related neurological conditions is resting-state perfusion or glucose metabolism imaging using SPECT and FDG-PET, respectively. which are highly inter-correlated[10,11] and reflect baseline local integrated synaptic activity[12]. Whether these imaging techniques have diagnostic value in CAA is however unknown, as to our knowledge no FDG-PET study in CAA is available in the literature, while the only published SPECT perfusion study was in non-ICH, mostly atypical presentation patients[13].

Performing both PiB and FDG-PET in suspected CAA patients is one option, but this would be time-consuming, costly and demanding on patients, and would increase radiation exposure. It would therefore be advantageous to obtain, from a single amyloid tracer administration, indexes of both perfusion and amyloid load[10]. It is well established that the early brain uptake of any radioligand provides a good estimate of tissue perfusion[14]. Accordingly, several authors have recently investigated the use of early-phase PiB data (typically obtained from 1 to 6 minutes after tracer administration) as a potential surrogate for perfusion or glucose metabolism mapping in normal subjects and AD[10,11], and as expected found an excellent correlation with FDG uptake obtained in the same subjects[10,11]. The same findings apply to AV–45, another amyloid PET tracer[15]. It therefore appears feasible to obtain an estimate of both neuronal dysfunction and amyloid burden by using early- and late-phase PiB data from the same PET session, which in turn might improve the diagnostic accuracy and particularly specificity of late-phase PiB in CAA.

Here we assessed early-phase ^11^C-PiB as an index of brain perfusion in sLICH patients diagnosed with probable CAA based on current Boston criteria, as compared to age-matched healthy controls. Our general aim was to determine if early-phase PiB could point towards potential diagnostic utility, particularly if combined with late-phase ^11^C-PIB.

Specifically we tested the following three hypotheses. Our first hypothesis is that whole-cortex early-PiB uptake will be reduced relative to aged HCs as a result of reduced perfusion from the widely distributed cortical (e.g., microbleeds, micro-infarcts, cortical siderosis) and white matter ischemic changes (undercutting the cortex) present in CAA[1]. Our second hypothesis is that the majority if not all the CAA patients will have individually reduced whole-cortex early PiB uptake while none of the aged HCs would, which would increase the diagnostic value of late-PiB uptake by combining both pieces of information.

However, as discussed extensively elsewhere[7], even if these hypotheses turned out to be correct, the above approach would not resolve the issue of how to interpret late-PiB positivity, which can reflect either CAA or AD as underlying Aβ-based pathology. We therefore also investigated whether early PiB uptake could help differentiate CAA from AD, and to this end also assessed a group of AD subjects. Specifically, our third hypothesis is that two cortical areas, namely the occipital cortex and the posterior cingulate cortex (PCC), will help towards this differentiation, for the following reasons: i) the PCC is among the regions earliest, most severely and most consistently hypoperfused/hypometabolic in mild-to-moderate AD[16,17], and accordingly PCC early-phase PiB uptake has been reported as significantly affected in this condition[10]; and ii) conversely, the occipital cortex is relatively preserved metabolically in mild-to-moderate AD[18,19], whereas it is particularly affected neuropathologically in CAA[20,21], and accordingly has been found on late PiB to be more affected in CAA than AD[5,7]. We therefore hypothesize that the PCC will have lower early PiB uptake in AD than in CAA, while the occipital region will have the opposite pattern, in turn allowing to optimally ‘contrast’ the two Aβ-related disorders using the occipital/PCC ratio.

## Subjects and Methods

### Subjects

The HCs and CAA patients used in this study have been described previously[7]. Eleven non-demented patients (9 men and 2 women; age: 70±7yrs) admitted for symptomatic lobar ICH and fulfilling current Boston criteria for probable CAA[2] were recruited through the Addenbrooke’s Hospital Stroke Unit or ICH clinic. Demographics and main clinical characteristics have been reported in detail previously[7]. They were all living independently with an MMSE score ≥23 (mean: 26.7±1.8), and all had ≥1 lobar microbleed and significant white matter ischemic disease (Fazekas score ≥2). They were recruited into this study at least one year after their last stroke.

Twenty medication-free healthy controls (HCs) with no memory or cognitive complaints and normal Mini-mental State Examination results (MMSE ≥29) were also recruited through advertisements posted at the University of The Third Age and the hospital advert board. This sample comprised of a ‘young’ subset of 10 HCs ≤55yrs which was used to derive normative values (see below), and a subset of 10 HCs aged 60 or older and matching in age the CAA group for comparison. One aged HC was excluded *post hoc* due to the presence of three lobar microbleeds on MRI as this might indicate incipient CAA. The final age-matched HC group therefore comprised 9 subjects (65±5yrs; MMSE range 29–30; NS and *p*< 0.001 relative to CAA, respectively).

Seven patients with mild-to-moderate AD were also recruited (male: 6; age: 65.5±5yrs, NS relative to CAA and HCs; MMSE: 22±6.7, *p* = 0.03 relative to CAA, *p*<0.001 relative to HCs); they were diagnosed with probable AD according to the NINCDS-ADRDA criteria [22]. None of them had lobar microbleeds on T2* 3T MRI.

The Cambridgeshire Regional Ethics Committee approved this study and all participants provided signed informed consent.

### Imaging Procedures: PET and MRI

The methodology used for [^11^C]PIB production and PET scanning in our centre has been previously reported[7]. Briefly, following a 15min Ge–68 transmission scan for attenuation correction, dynamic PET emission scanning in 3D mode was undertaken for 90 minutes following injection (58 frames: 18×5s, 6×15s, 10×30s, 7×1min, 4×2.5min, 13×5min). The protocol also included a 3D-volume T1-weighted MPRAGE acquired on a 3T-MRI for co-registration, spatial normalization and regions-of-interest (ROIs) purposes. Standard GRE and FLAIR were also acquired for this study.

### Image Analysis and early-phase PiB data

The general procedures have been previously published in detail[7]. First, early-phase PiB images were generated by collapsing all decay-corrected emission images acquired from 1 to 6 min after ^11^C-PiB injection[10], and late-phase PiB by using the 35–90mins dynamic dataset. The PET images were then co-registered to the MPRAGE dataset as described[7]. The MRI (and coregistered PET data) were spatially normalised to the MNI/ICBM152 2009a T1-weighted template using Symmetric Image Normalisation (SyN), an advanced algorithm reducing risk of normalization errors[23]. This accurate spatial normalization was also necessary to allow the use of standard space ROIs[24]. Following segmentation of the T1 dataset and smoothing to approximate the PET resolution, grey matter (GM) ROIs were defined as the intersection between Automated Anatomic Labeling (AAL) ROIs[25] and ≥65% thresholded GM segments. To mitigate the effects of cortical atrophy in this aged population, the PET data were corrected for partial volume effects from cerebral spinal fluid (CSF), using Meltzer et al two compartment method[26]. This uses tissue class segments from MR to model the contribution of brain and CSF to each PET voxel value. The segments (grey matter, white matter and CSF) were determined from the MPRAGE using SPM2. To accurately model the contributions of brain (grey matter + white matter) and CSF to a voxel at the resolution of PET (6.8mm FWHM isotropic), the MR segments were smoothed by a 6.7mm FWHM isotropic Gaussian. Within the AAL ROIs, each voxel was corrected for CSF contamination by dividing each PET voxel value by the corresponding voxel value of (1-sCSF), where sCSF denotes the fractional probability of the voxel being CSF. This voxel-wise CSF correction was applied within all ROIs, including the reference region ROI. Finally, for each ROI the mean CSF corrected voxel value within the ROI was used to determine the standardized uptake value (SUV).

Based on our objectives and main hypotheses, a whole cortex ROI was computed as the sum of all AAL cortical ROIs. In addition, as per our third hypothesis, the occipital and posterior cingulate GM ROIs were also obtained. For each patient, any ROI directly affected by the lobar ICH was excluded *a priori* from the analysis. Early-phase PiB SUVs were obtained for the whole-cortex (weighted average), occipital and PCC ROIs, and normalized by the CSF-corrected SUV of the cerebellar vermis[27] (defined as the sum of all vermis ROIs from AAL), to yield SUV ratio (SUVR) values. Then, the ROI data were averaged across the right and left hemispheres prior to statistical analysis. Regarding late-phase PiB, and as detailed elsewhere, distribution volume ratios relative to cerebellum were obtained for the same ROIs using the 35–90mins CSF-corrected average ROI data[7].

### Data analysis

To address our first hypothesis (see Introduction), we compared the mean whole cortex early-phase PiB SUVR values between CAA and aged HCs. To test our second hypothesis, we then assessed for each subject (CAA and aged HCs) their positive or negative status relative to the 95% lower confidence limit derived from the whole cortex early PiB uptake in the younger HC group, and subsequently combined this information with similar information derived from late-phase PiB data to see if doing so effectively increases the sensitivity and specificity of the latter. Finally, to test our third hypothesis, we compared the occipital and PCC GM ROIs as well as the occipital/PCC ratio between the CAA and AD groups, and relative to the aged HCs.

### Statistical Analysis

ROI SUVR values were first compared between the CAA and aged HC groups, using a two sampled t-test. We then determined the sensitivity and specificity of whole-cortex early-phase PiB uptake as a potential diagnostic test for CAA vs age-matched HCs. The third analysis was carried out to compare the occipital and PCG data among the three groups, using ANOVAs for each ROI with Groups as fixed factor. Tukey’s post-hoc tests were then applied if significant effects were present. Finally, the occipital/PCC ratio was compared between the CAA and AD groups using the non-parametric Mann-Whitney test to accommodate for the intrinsic non-linearity of ratios. Statistical significance was defined as 2-tailed *p*<0.05.

## Results

### Whole-cortex analysis: early-phase PiB

The whole cortex early-phase PiB SUVR was significantly lower in the CAA than the aged HC group (0.96 ± 0.08 vs 1.04 ± 0.05, respectively; p = 0.034).

The lower 95% confidence limit for Whole Cortex SUVR derived from the subgroup of young healthy controls (normally distributed dataset) was 0.94. No aged control fell below this cut-off, while 6/11 CAA patients did (sensitivity = 55%, specificity = 100%, predictive positive value = 100%, predictive negative value = 64%).

### Added value of whole cortex early-phase PiB onto late-phase PiB

Table 1 shows the individual findings with both late- and early-phase PiB. The only late-phase PiB negative CAA patient fell above the early-phase PiB 95% cutoff, i.e., within the normal range. With this CAA patient being negative with both early- and late-phase PiB, the combined sensitivity of the two PiB phases added together was 91%, i.e., unchanged from late-phase PiB. Since all aged HCs were early PiB negative but 4/9 were late PiB+, the combined specificity also remained at 55%. Note that 5 CAA patients were also late-PiB+ but early PiB-. Table 2 shows how combining early- and late-phase PiB could help in improving the diagnostic value of PiB-PET for CAA.

**Table 1:** Individual whole-cortex SUVr findings obtained with early- and late-phase ^11^C-PiB-PET in age-matched healthy controls (N = 9) and CAA patients (N = 11), relative to the 95% confidence limit determined in young healthy controls (see Methods for details).

	Late PiB	Early PiB
**Aged controls**		
**1**	+	-
**2**	-	-
**3**	-	-
**4**	+	-
**5**	-	-
**6**	-	-
**7**	+	-
**8**	-	-
**9**	+	-
**CAA patients**		
**1**	+	-
**2**	-	-
**3**	+	-
**4**	+	+
**5**	+	+
**6**	+	+
**7**	+	-
**8**	+	+
**9**	+	+
**10**	+	-
**11**	+	-

**+** Positive (i.e., outside the normal range).

- Negative (i.e., within the normal range).

**Table 2:** Diagnostic paradigm using the combined results from both early- and late-phase whole-cortex ^11^C-PiB-PET in suspected CAA-related lobar intra-cerebral hemorrhage.

Late-phase ^11^C-PiB	Early-phase ^11^C-PiB	Interpretation
**+**	**+**	CAA likely^**&**^
**-**	**-**	CAA unlikely but possible
**-**	**+**	CAA unlikely but possible
**+**	**-**	Not informative

&: However does not formally differentiate between underlying CAA and AD.

### Distinguishing CAA from AD

**Fig 1:**
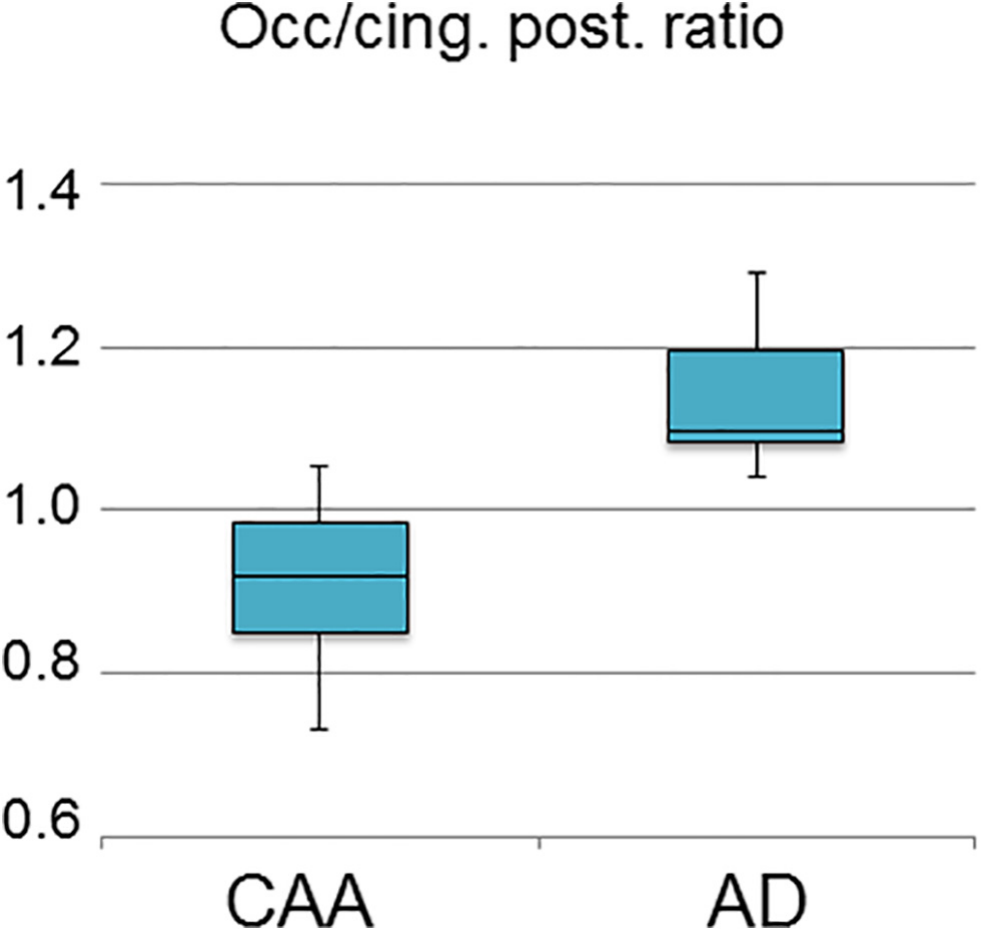
Box plot showing the highly significantly lower early-phase ^11^C-PiB occipital/posterior cingulate SUVR ratio in CAA patients (n = 11) as compared to Alzheimer’s disease (AD; n = 7) (p = 0.002, Mann-Whitney non-parametric test).

The mean (and 1SD) SUVR values for the occipital and PCC ROIs are shown in Table 3. The within-ROI ANOVA showed significant (*p*<0.05) differences among the three groups for both ROIs. The *post-hoc* tests comparing among the three patient groups revealed results entirely consistent with our hypotheses. Thus, the CAA group had significantly lower occipital SUVR than both the age-matched healthy controls and the AD groups, while the AD group did not differ from the healthy controls. Likewise, regarding the PCC, only the AD group had significantly lower early PiB uptake as compared to the aged controls. Accordingly, the Occipital/PCC ratio was strongly significantly lower in the CAA relative to AD group (*p* = 0.002, Mann-Whitney) (Table 3, Fig 1). As this ratio was also significantly higher in AD relative to HCs but not different between CAA and HCs, it specifically discriminated CAA from AD.

**Table 3:** SUVR data (mean ± 1SD) for the three selected ROIs among the three groups of subjects, namely aged-matched healthy controls (HC; n = 9), probable cerebral amyloid angiopathy (CAA; n = 11) and probable Alzheimer’s disease (AD; n = 7). For the ROI data, statistical comparison among the three groups was carried out using ANOVAs followed by post-hoc Tukey tests whenever the ANOVA was significant. For the occipital/PCC ratio, the comparisons were made using the non-parametric Mann-Whitney test. See Results for details.

	Occipital cortex	Posterior cingulate cortex	Occipital/posterior cingulate ratio
**HC**	1.02 ± 0.04	1.04 ± 0.05	0.98 ± 0.03
**CAA**	0.94 ± 0.05	0.99 ± 0.05	0.95 ± 0.06
**AD**	1.03 ± 0.06	0.98 ± 0.04	1.07 ± 0.05
1. CAA vs HC	*p* = 0.018	*p* = 0.112	*p* = 0.30
2. CAA vs AD	*p* = 0.006	*p* = 0.766	*p* = 0.002
3. AD vs HC	*p* = 0.806	*p* = 0.046	*p* = 0.001

**Fig 2:**
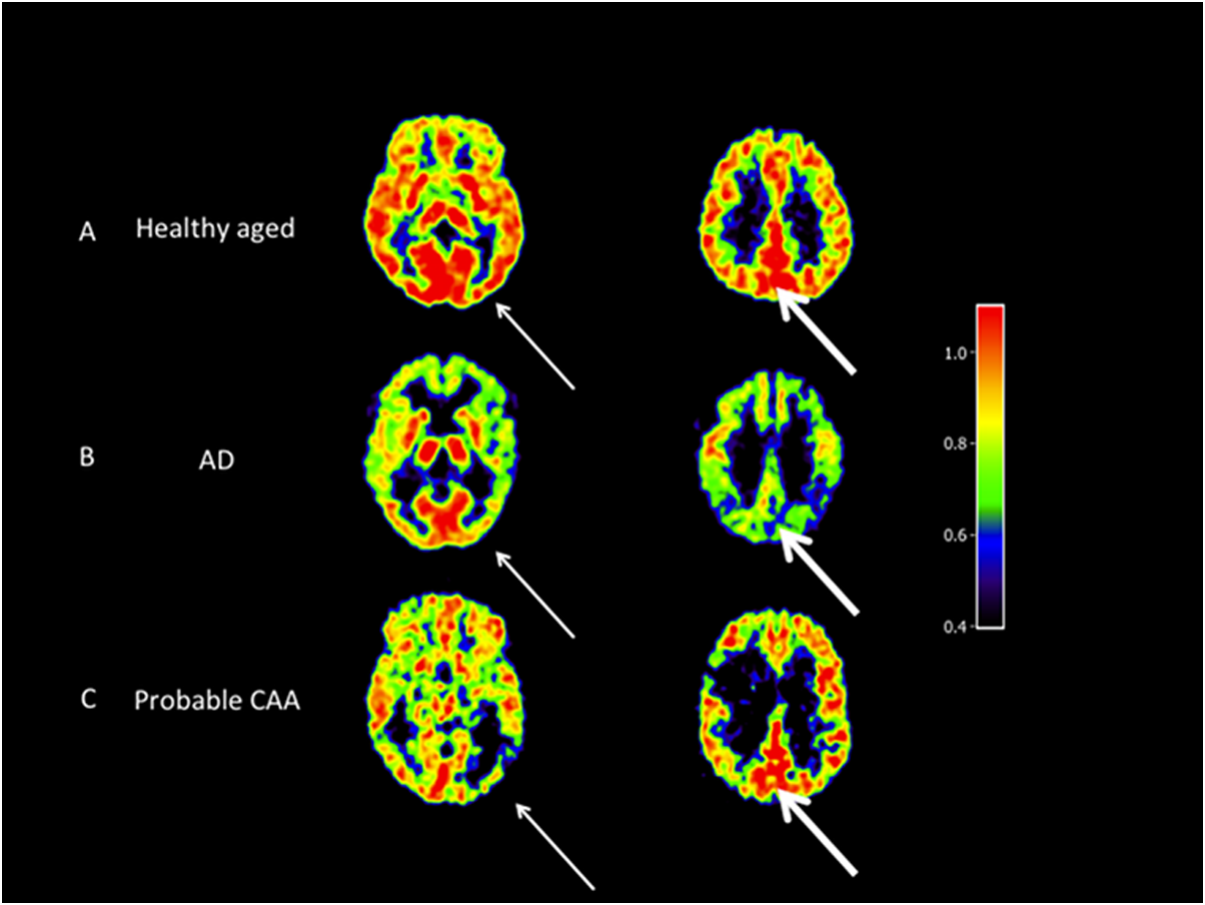
Representative early-phase PiB SUVR axial images (normalized to the subject’s vermis value) for the basal ganglia (left column) and centrum semiovale level (right column) from one subject each from the age-matched healthy controls (A), AD (B) and probable CAA-related sLICH groups (C). The thin arrows point to the occipital region, showing the typical low uptake in the CAA profile as compared to age-matched controls and AD. The bold arrows point to the posterior cingulate area showing reduced uptake in AD as compared to age-matched controls and CAA. SUVRs were obtained from early-phase ^11^C-PiB-PET using the cerebellar vermis as reference ROI (see Methods).

Fig 2 displays typical imaging findings in one subject from each group.

## Discussion

Consistent with our hypothesis, whole-cortex early-phase PiB uptake was significantly decreased in the CAA group relative to aged-matched controls, suggesting that as predicted overall cortical perfusion is reduced in CAA. No aged control fell below the 95% confidence limit for whole-cortex early-phase PiB derived from young controls, resulting in a 100% specificity. However, only 6/11 CAA patients were early-PiB positive, resulting in a sensitivity of 55%. Combining the early- and late-phase whole-cortex PiB data did not nominally improve sensitivity and specificity, but since, contrary to late-phase PiB, no aged HC was early-phase PiB positive while both early- and late-phase PiB positivity was present only in CAA patients, this double-positivity points to a very high suspicion of underlying Aβ-related clinical disorder, i.e., CAA or AD (Table 2). In order to try and differentiate between these two possibilities, we then show that, as predicted, the early-phase PiB occipital and posterior cingulate uptakes, and more specifically the occipital/PCC ratio, have significant diagnostic potential. Thus, early-phase PiB may provide substantial added value to late-phase PiB in diagnosing CAA relative to both normal aging and AD.

Our finding of significantly reduced whole-cortex early-phase PiB uptake in probable CAA is novel. To the best of our knowledge, there is no published study of FDG uptake in CAA to compare our results to. Regarding perfusion, a brain SPECT study reported focal hypoperfusion in various locations in CAA but this was in non-ICH, atypical presentation patients, many with mixed diagnoses[13]. As previous studies have documented that early-phase PiB strongly correlates with FDG uptake[10,11], it can be assumed that the early-phase PiB decreases observed here represent neuronal/synaptic dysfunction[12]. This cortical dysfunction may reflect the cortical pathology present in CAA, which includes microinfarcts, microbleeds and cortical siderosis, as well as cortical deafferentation resulting from white matter ischemic damage[1,20,21,28,29]. However, an additional potential mechanism involves cortical ‘intra-hemispheric’ diaschisis[30] from the lobar hematoma[31,32], the latter being by definition present in all our patients. Chronic diaschisis mainly reflects Wallerian fiber degeneration without clear cognitive counterpart[30,33]. To address this hypothesis, we repeated *post hoc* the analysis after removing all AAL ROIs from the hemisphere affected by ICH across the whole sample. Doing this removed the statistical significance, suggesting this mechanism plays a major role in the observed reduction in whole-cortex early-phase PiB uptake. Note however that this post-hoc analysis did not remove the strong difference in occipital/posterior cingulate ratio between the CAA and AD groups, which remained highly significant (*p*<0.001).

Early-phase PiB SUVR was found to have a sensitivity of 55% and a specificity of 100% for CAA vs aged HCs. Although preliminary as based on a small sample and not generalizable to other presentations of probable CAA, these results are nevertheless of interest because using late PiB uptake in the same sample, the opposite results were found, namely a very high (91%) sensitivity but a low specificity (55%) due to the occurrence of late-phase PiB positivity in four of the nine healthy elderly subjects[7] (Table 1). Importantly, all of the aged HCs, including all four late-phase PiB+ subjects, were early-phase PiB negative. These findings are consistent with the recent report of only mild, albeit statistically significant, cortical FDG uptake decrease in late-phase PiB+ healthy elderly[34]. However, the only CAA patient who was late-phase PiB negative was also early-phase negative, meaning that the sensitivity of combined early- and late-phase PiB remained at 91%. Note however that this patient’s late-phase PiB uptake fell just below the normal 95% confidence limit[7].

Table 2 shows the different situations that can be encountered combining both early- and late-phase PiB when assessing a lobar ICH patient suspected of CAA, and their diagnostic implications. Given that no healthy aged control was both early- and late-phase PiB positive, adding early-phase PiB clarifies the previously highlighted ambiguity of late-phase PiB+[7]. In other words, the situation of both early- and late-phase PiB positivity in lobar ICH patients suspected of CAA is strongly suggestive of underlying amyloid-related clinical disorder, i.e., CAA or AD. Because of the rare occurrence of negative late-phase PiB in our CAA sample, namely 1/11 cases, late-phase PiB negativity makes the possibility of CAA unlikely but does not exclude it, regardless of the early-phase findings. Finally, late-phase positivity with early-phase negativity does not provide any useful information, i.e., the interpretation remains ambiguous.

In order to clarify the CAA vs AD ambiguity in case of late-phase positivity, we assessed whether early-phase PiB can help to differentiate between these two possibilities. As predicted, the early-phase PiB occipital uptake and the occipital/PCC ratio were both markedly lower in CAA relative to AD (Table 3). Thus, in patients with lobar ICH and late-phase PiB positivity, the early-phase occipital/PCC ratio might help rule out AD and hence strengthen the suspicion of CAA. Further studies in larger cohorts should assess the sensitivity and specificity of this ratio.

The only previous study reporting baseline occipital perfusion in probable CAA-related ICH used MR-based arterial spin labelling and found no difference in absolute blood flow[35], but relative perfusion data was not reported, making the comparison to our study difficult.

One potential confounder in the above paradigm is Lewy Body disease (LBD), a neurodegenerative disorder partly overlapping with AD neuropathologically, where amyloid PET is frequently positive and that is characterized by reduced occipital perfusion and FDG uptake, as well as positive DaT-scan[19,36]. However, LBD is unlikely to cause confusion in practice given the quite specific clinical context.

In the present study we corrected the regional PET data for cortical atrophy, using the MR-derived fraction of CSF in each voxel to increase the voxel PET count, according to the widely used Meltzer method[26,37,38]. This is recommended when studying aged populations with potentially varying degrees of global and regional atrophy as here, in order to prevent spurious findings from artefactually reduced PET counts. However, applying the Meltzer correction voxel-wise can increase data noise due to the (1/x) type of calculation, potentially causing bias. However, the procedure employed in the present study was designed to constrain this risk. First, we selected for analysis only those voxels whose grey matter (GM) fraction was ≥65%, so that the largest possible CSF contribution was 35%, and hence the largest CSF correction scaling factor applied to a voxel was 1.538 [i.e. 1/(1–0.35)]. Second, prior to applying this correction, the GM and CSF segments were smoothed down to PET resolution. Thirdly, the values we used for the present study were the means calculated for the whole ROI, rather than single voxel data. Nevertheless, we repeated *post-hoc* the analysis without CSF correction, which did not substantially change the overall results. Notably the occipital/PCC ratio, which is largely independent of CSF correction, continued to highly significantly discriminate between CAA and AD (data not shown).

Our findings are based on small samples and lack a direct validation against FDG or perfusion PET, and are therefore preliminary. Nonetheless, they do support the idea that combining both early- and late-phase ^11^C-PiB may be useful in the diagnostic work-up of ICH patients suspected of CAA, particularly in case of late-phase PiB positivity. Further studies in larger cohorts of probable CAA patients, if possible including pathological diagnosis, are required.
